# Managing anxiety and fear associated with HPV vaccination in Adolescents

**DOI:** 10.1192/j.eurpsy.2026.11178

**Published:** 2026-07-31

**Authors:** N. Bouayed Abdelmoula, B. Abdelmoula

**Affiliations:** LR23ES07 1Genomics of Signalopathies at the service of Precision Medicine, Medical University of Sfax, Sfax, Tunisia

## Abstract

**Introduction:**

Vaccination against the human papillomavirus (HPV) is essential to prevent HPV-related cancers, but is often accompanied by anxiety and fear in adolescents. These psychological barriers can have a negative impact on the adoption and completion of vaccination. Understanding the causes and mitigation strategies of vaccination-related distress is essential to improve compliance.

**Objectives:**

The aim of this study is to explore the causes of HPV vaccination-related distress among adolescents and the potential mitigation strategies.

**Methods:**

We conducted a comprehensive review of the scientific literature using scientific databases and key words including HPV, Vaccination, Psychiatry.

**Results:**

Anxiety and fear in adolescents are mainly motivated by anticipatory distress, misinformation, fear of pain and rumors about the side effects of vaccines. Without intervention, these fears contribute to distress and avoidance of doses. Evidence indicates that pre-vaccination education, open parental communication, peer encouragement, distraction techniques during vaccination and health personnel trained in cognitive behavioral strategies significantly reduce anxiety levels and improve vaccination experiences.

**Conclusions:**

The psychological burden surrounding the HPV vaccination is manageable thanks to targeted psychosocial interventions. The integration of education and emotional support before and during vaccination promotes a more positive attitude, reduces refusals motivated by fear and improves adherence to the vaccination schedule. Proactive strategies involving adolescents, families, peers and health care providers are essential to alleviate anxiety and fear related to HPV vaccination. Such comprehensive approaches can promote greater use of the vaccine and contribute to effective prevention of HPV-related cancer. In summary, the main cause of persistent fear is the lack of adequate information and psychosocial support. Programs that integrate education, parental participation, peer encouragement and qualified staff significantly reduce anxiety and promote a more positive vaccination experience.

**Disclosure of Interest:**

None Declared

